# Case Report: TNF-Alpha Inhibitors to Rescue Pregnancy in Women With Potential Pregnancy Loss: A Report of Ten Cases

**DOI:** 10.3389/fimmu.2022.900537

**Published:** 2022-05-25

**Authors:** Zixing Zhong, Yuhan Wang, Guiqin Wang, Feifei Zhou

**Affiliations:** ^1^ Center for Reproductive Medicine, Department of Obstetrics, Zhejiang Provincial People’s Hospital (Affiliated People’s Hospital, Hangzhou Medical College), Hangzhou, China; ^2^ The First Clinical College of Zhejiang Chinese Medical University, Hangzhou, China; ^3^ Fertility Center of Melinda Women and Children’s Hospital, Dalian, China; ^4^ Center for Reproductive Medicine, Department of Traditional Chinese Medicine, Zhejiang Provincial People’s Hospital (Affiliated People’s Hospital, Hangzhou Medical College), Hangzhou, China; ^5^ Zhejiang Women’s Hospital, School of Medicine, Zhejiang University, Hangzhou, China

**Keywords:** miscarriage, case series, hCG (human chorionic gonadotrophin), TNF – α, pregnancy-rescue management, TNFi

## Abstract

Miscarriage poses a significant threat to pregnant women globally. Recurrent miscarriages or potential poor embryonic development indicated by early drops in serum human chorionic gonadotrophin (hCG) are even more catastrophic for pregnant women. However, these patients receive either individualized medical intervention supported by limited evidence or no treatment at all. In this study, we report ten patients who shared at least one episode of an early decline of hCG in the first trimester and were treated with compassionate use of tumor necrosis factor-alpha inhibitor (TNFi). They were then followed up regularly with caution. Their hCG trajectory all resumed a normal pattern within one week and the obstetric outcomes were promising. No adverse fetal, neonatal, or maternal health issues have been observed. This case series supports current safety evidence of TNFi and provides new insight into its use in pregnancy when the embryo is in danger. Further well-designed clinical trials should be carried out to consolidate the evidence.

## Introduction

Miscarriage is defined as the loss of pregnancy before viability. It causes significant psychological and economic damage to the family and society (1). Adopting active strategies to prevent miscarriage, especially in women with a history of miscarriage, has been called for by Prof. Coomarasamy and others in recent years (2). However, there are few evidence-based guidelines for healthcare providers to prevent miscarriage.

hCG is a glycoprotein with a molecular weight of 36,000 to 40,000 Dalton. It is almost exclusively produced by trophoblast cells and placenta after placental formation (3). The trajectory of serum hCG results from serial tests in early pregnancy demonstrates excellent diagnostic and prognostic value across women with different ethnic backgrounds and age groups (4). This pregnancy hormone proliferates in early pregnancy, doubling its level every two days, reaching its peak at 50,000 to 100,000 mIU/mL between 8 and 10 weeks, and then gradually declines until delivery (3). When the hCG increases less than 50% in 48 hours, only a minority of embryos can survive (5). An early decline of hCG generally indicates irreversible poor embryonic outcomes and expectant management is often advised (6, 7). The up-to-date evidence has caused concern for women as even one previous miscarriage can increase the risk of recurrent miscarriages in subsequent pregnancies (1). Therefore, safe and effective measures should be considered to help women. At present, there is sparse evidence that can provide guidance when pregnancy is in danger.

Tumor necrosis factor-alpha (TNF-α) is an inflammatory cytokine belonging to Th1 lymphocytes. TNF-α blockers inhibit the effect of TNF-α. The agents were used in rheumatoid arthritis (RA), ankylosing spondylitis, inflammatory bowel disease (IBD), and psoriasis (8). It has also been shown to play a vital role throughout pregnancy (9, 10). The use of TNF-α anti-therapy (TNFi) in pregnancy is supported by The British Society for Rheumatology and British Health Professionals in Rheumatology (BHPR), European Alliance of Associations for Rheumatology (EULAR), American College of Rheumatology (ACR), and American Gastroenterological Association (AGA) as guidelines on prescribing various medications in pregnancy (11–16). However, this therapy has generally limited to pregnant women complicated with autoimmune diseases until recent years, when immunosuppressive medication has been suggested to play an essential role in recurrent miscarriages (17).

In this case series, we report the trial of TNFi-led pregnancy-rescue management (TPRM) to help women with early signs of miscarriage. Most of the women in our study had a history of recurrent pregnancy loss according to the definition from the European Society of Human Reproduction and Embryology (ESHRE) guideline (18). Some cases were complicated with complex autoimmune disorders, thrombophilias, or imbalanced cytokines (19). All women who had received treatment resumed the growth of hCG and enjoyed ideal obstetric outcomes later on.

## Case Descriptions

The basic characteristics include women’s age, body mass index (BMI), previous obstetric background, and obstetric outcomes in current pregnancies. Details can be seen in **Tables 1**, **2**. All women are Han Chinese (the major ethnic group in China). The average age was 32.40 years (27-40), while the average BMI was 24.4. 4/10 were overweight (BMI > 24.99). All ten women had been offered preventative therapy with low-dose aspirin (LDA), low molecular weight heparin (LMWH), or progesterone therapy in the early stage due to previous obstetric histories. Their hCG growth followed the standard trajectory before the unexpected decline in the first-trimesters.

**Table 1:** Demographic data and main disorders complicating pregnancy in the case series.

Case	Age	BMI	Maternal condition in the current pregnancy			
			Endocrinology	Autoimmune disorder	Hematological disorder	Anatomy
1	27	23.3	–	–	Thrombophilia	Class U4/Hemi Uterus^*^
2	34	24.7	PCOS	NOT tested	–	–
3	36	30.0	Hashimoto IR	Non-classical APS	Protein C antibody (+)	–
4	29	18.8	Hashimoto	–	Thrombophilia	–
5	32	23.0	–	APS (β2GP1 > 100)	Thrombophilia	–
6	40	29.4	–	–	–	–
7	31	29.3	PCOS	TNF-α 15.9	–	–
8	28	20.7	–	–	–	–
9	33	18.6	–	TNF-α 300+	–	–
10	34	26.2	–	–	–	Fibroids

^*^The Class U4/Hemi a Uterus is defined as the hemi uterus with a rudimentary cavity, which is the case of patient No.1. The diagnosis is made as per the ESHRE/ESGE consensus in 2013 (20).

**Table 2:** Previous obstetric history and current pregnancy outcomes.

No.	Previous obstetric history	Mode of conception	TNFi therapy	Mode of delivery	GA (wks)	BW (g)
1	FL1 EP1*	NC	Adalimumab*1 dose	CD	37^+4^	3220
2	Para1	NC	Adalimumab*1 dose	CD	38^+2^	3170
3	MC3	ART-FET	Adalimumab*2 doses	CD	39^+5^	3850
4	MC1	NC	Certolizumab pegol*1 dose	Ongoing pregnancy	31	–
5	RIF7	ART-FET	Adalimumab*1 dose	Ongoing pregnancy	33	–
6	MC2	ART-FET	Adalimumab*1 dose	CD	38^+6^	3700
7	Para1 EP2 MC1	NC	Adalimumab*3 doses	CD	39^+1^	3750
8	–	NC	Adalimumab*1 dose	VD	40^+0^	3550
9	MC4^※^	NC	Adalimumab*1 dose	CD	37^+4^	3000
10	MC2	NC	Adalimumab*1 dose	CD	38^+2^	2780

GA, Gestational age at birth according to CRL measured at NT scan for women with natural conception, while gestational age was calculated according to their day of embryo transfer for ART women; FL, Fetal loss (> 10wks); MC, Miscarriage (<10wks); RIF, Recurrent implantation failure; EP, Ectopic pregnancy; NC, Natural conception; ART, Artificial reproductive technology; FET, Frozen embryo transfer; BW, Birthweight; CD, Cesarean delivery; VD, Vaginal delivery.

^*^Pregnancy in rudimentary horn.

^※^Four miscarriages without explanatory causes after systematic investigations.

## Diagnostic Assessment

**Figure 1:**
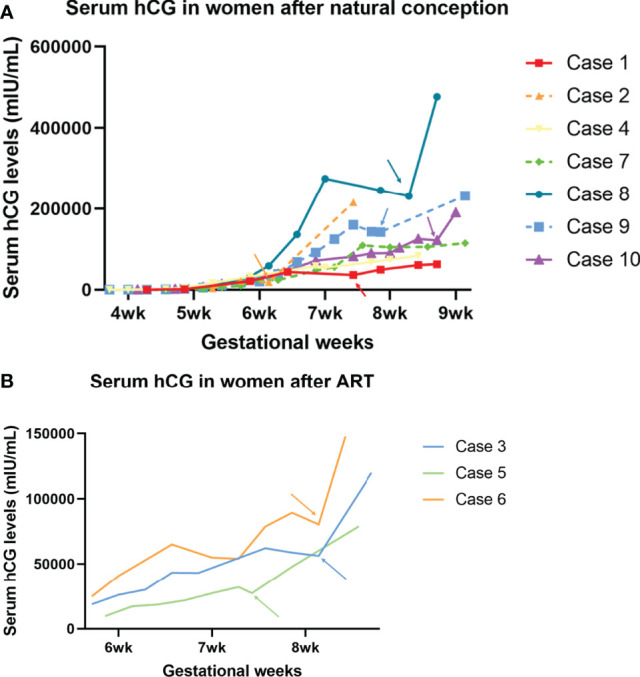
Serum hCG trajectory of each of the ten cases. The generated curve of serial hCG values of women who present early decline in the first trimester in the current pregnancies. **(A)** The subgroup of women following natural conception (n=7). **(B)** Subgroup of women following ART (n=3). Arrows with corresponding colors indicate the timing of administration of TNFi.

The overall serial serum levels of hCG are shown in **Figure 1**. Detailed data of serial serum hCG can be seen in the supplementary file. All of the premature decline events occurred from 40 days onwards, with the hCG value at more than 20,000mIU/ml on the test day. Most (9/10 cases) early declines occurred between six and eight gestational weeks. They were then rescued with TNFi (certolizumab for case No. 4 and adalimumab for the other nine cases) and followed up on an average of 3.6 days (1-9 days) after injections. Case No. 3, 6, and 7 experienced two episodes of the early drop of hCG in the first-trimester. As hCG trajectories in early pregnancies are different between natural pregnancy and assisted reproduction, cases are further sub-classified according to their mode of conception.

### Case No. 3, 5, and 6

Three out of the ten cases were pregnancies following ART. Case No. 3 and 6 were diagnosed with unexplained infertility with at least two episodes of early embryonic failures after ART, while case No. 5 was affected by tubal factors. In case 3, upon the first episode, when hCG declined from 43,271 to 43,185, the patient had concurrent minor vaginal bleeds, indicating a threatened miscarriage. She received 1g tranexamic acid twice daily and a single dose of adalimumab (40mg) for one day and the bleeds stopped. A further decline on Day 55 prompted a second off-label use of adalimumab. Six days after injection, the serum hCG level went back on track (hCG declined from 62,164 on Day 53 to 58,786 on Day 55, then climbed to 120,006 mIU/mL on Day 61). In contrast, case No.6 received only one dose of adalimumab despite having experienced two episodes of early declines of hCG. Case No. 5 had only experienced one episode of hCG early decline and received one single dose of adalimumab on that day. She is now 33wks and is followed up regularly in the antenatal clinic and no stillbirth or any other adverse fetal or maternal outcome has been reported.

### Case No. 1, 2, 4, 7, 8, 9, and 10

Among the seven patients who were conceptualized naturally, case No. 9 and 10 had two and four previous spontaneous miscarriages, respectively. Case No. 2 and 7 had a successful obstetric history before the current pregnancy. Case No. 8 was not complicated with pre-existing maternal medical conditions, nor did she have a previous history of miscarriage. However, she suffered from a long-lasting early decrease of hCG for more than ten days, suggesting an adverse embryonic outcome. A similar case was observed in No. 4, who is now 31wks, after being offered a single dose of certolizumab pegol (200mg) at 7wks when hCG fell from 58,016 to 54,438 mIU/mL the following day.

Eight out of the ten women have delivered so far. The average birth weight was 3377.50g (2780-3850g), which fell into the normal reference range. Despite uneventful pregnancies after the rescue therapy, we identified a disproportionally high cesarean section rate in the study group. Case No. 8 is the only one so far who has delivered vaginally. Case No. 3 and 6 had cesarean for fetal distress in labor, possibly resulted from oligohydramnios (not an absolute indication for cesarean but could lead to increased emergency cesarean delivery in labor). Case No. 2 and 7 had CS (C-sections) indicated for previous CS delivery, a major obstetric indication in China. The other three cases had requested a cesarean delivery (also known as CDMR, cesarean delivery on maternal request) in spite of any obstetric indications. This partly reflects China’s high rate of CS in the low obstetric risk population (21).

## Discussion

In most cases, women would generally be offered expectant management if a spontaneous miscarriage is highly anticipated. However, the novel use of TNF-α blocker therapy has rendered promising obstetric outcomes in all patients. Safety concerns have also been addressed as no maternal-fetal or neonatal adverse consequences are reported in any patient. To our knowledge, this is the first report on the compassionate use of TNF-α blocker therapy to rescue endangered pregnancies.

There are guidelines for pre-conceptional and prenatal care in China, but they aim to serve the general population (22). Anxious women in China usually tend to make more frequent antenatal visits to request more investigations. Blood tests are more frequently performed in early pregnancies than others, such as ultrasound scans, as the former are usually more readily accessible in the antenatal clinics.

Highly individualized therapies are currently practiced in response to unexpected results revealed in early pregnancy, particularly in women with a history of multiple pregnancy losses but without definite explanatory causes for their recurrent pregnancy losses (2, 23). We agree that it is better to do something safe that may be effective than do nothing. The pre-condition of our strategy is that the embryonic pole (ideally with an active fetal heartbeat) must be seen in all women *via* ultrasound despite falling hCG levels.

The maternal immune system strikes an intricate balance between supporting embryonic development and maintaining immune responses in pregnancy. A dysregulated maternal immune system in pregnancy can affect obstetric outcomes (24, 25). TNF-α, the multifunctional T-helper 1 (Th1) cytokine, influences hormone synthesis, placental construction, and embryonic development (26). The altered expression of TNF-α can cause recurrent miscarriages and pre-eclampsia (27, 28). The pivotal role of pro-inflammatory cytokines and innate immunity during early pregnancy paved the way for efficient and safe treatments such as TNF-α antagonists (29).

We used adalimumab (Humira) and certolizumab pegol (Cimzia) in all cases. Adalimumab is a monoclonal IgG1 antibody, which is actively transported across the placenta by the neonatal Fc receptor (FcRn) (30). Certolizumab pegol (CZP) neutralizes both the membrane and the soluble form of human TNF-α in a dose-dependent manner. Its unique structure may prevent fetal exposure to the drug during pregnancy (31, 32). Both of them can be safely used in pregnancy to treat some autoimmune diseases and have recently been introduced to clinical trials on refractory recurrent spontaneous miscarriages (23, 29, 33). Despite little experience in applying CZP for pregnancy rescue therapy, we will further consider its use because its unique structure could avoid placental transfer, making it a better, safer choice in pregnancy.

In our case series, we proposed a different therapeutic strategy only when pregnancies were endangered. All cases were closely monitored with serial serum hCG tests before and after adalimumab or certolizumab injection. The trajectory of hCG resumed a normalized pattern day 4.9 on average. In contrast, it takes about two weeks for TNF-α blockers to relieve the symptoms in patients with diseases such as RA. Hence, we assume from the data that it takes much less time for the agents to modulate the microenvironment in the maternal-fetal interface (34).

Compared with low-dose aspirin and LMWH or progesterone used in other clinical trials to prevent miscarriages in recent years, we use the TNFi as the primary therapy for rescuing pregnancies instead of preventing miscarriages (2, 17, 23, 29).

With this trial, we are the first to report a novel strategy with the use of TNFi that could save pregnancies with potential risks of miscarriage. It offers insights to other healthcare providers when they have patients with reduced hCG in early pregnancy. This may benefit pregnancy in the specific situation without compromising maternal and neonatal health. However, there are some limitations to our study. First, the number of this series is too small to reach a consolidated conclusion. Second, all women have been offered a range of medications rather than TNFi-only therapy. The use of medicines could compromise the pharmacological effect of TNF-α blockers in rescuing pregnancy. Third, although all early declines of hCG were observed in the same hospital, human factors could add to inaccurate testing of serum hCG levels in early pregnancy. However, even if we consider this, a sub-optimal growth is still considered to indicate possibly poor embryonic development.

## Conclusion

Our study has demonstrated that TNFi can reverse potentially poor pregnancy outcomes in the first trimester. However, it can only be used in the context of a clinical research setting, and further trials and experiments should be carried out to confirm the pharmacological effect of TNFi in rescuing pregnancies at risk.

## Patient Perspective

Case No. 1: I agree to the novel use of TNFi-led therapy for my specific situation and to report any problems for a better medical understanding and improvement of treatment options for women with a potential risk of miscarriage. I hope this enables the doctors to help pregnant women with safe and effective treatment. I am now very happy with my newborn baby.

Case No. 2: I agree to report on my case, the pregnancy, and the data of my baby. I hope this article can help other women like me get appropriate therapy for their threatened miscarriages.

Case No. 4: I agree to report my particular condition for a better medical understanding and improvement of treatment options for pregnant women whose hCG declines in early pregnancy. At present, I am 31 weeks pregnant. The antenatal examination and investigations have been normal and the fetus is developing well. I hope that this report could help more patients who share similar conditions with me in China and other countries worldwide.

## Data Availability Statement

The raw data supporting the conclusions of this article will be made available by the authors, without undue reservation.

## Ethics Statement

The studies involving human participants were reviewed and approved by the Ethics Committee of Zhejiang Provincial People’s Hospital. The patients/participants provided their written informed consent to participate in this study. Written informed consent was obtained from the individual(s) for the publication of any potentially identifiable images or data included in this article.

## Author Contributions

ZZ and FZ developed the idea for this study. ZZ wrote the manuscript and FZ reviewed it. YW and GW are involved in the diagnostic and therapeutic care and follow-up of the patients. YW and ZZ are also responsible for the tables and figures formation and revision. All authors contributed to the article and approved the submitted version.

## Funding

This study is co-supported by Zhejiang Provincial Project for Medical and Health Science and Technology (Grant no. 2022503241) and Zhejiang Provincial Project for Medical Education (Grant no. Y202146113).

## Conflict of Interest

The authors declare that the research was conducted in the absence of any commercial or financial relationships that could be construed as a potential conflict of interest.

## Publisher’s Note

All claims expressed in this article are solely those of the authors and do not necessarily represent those of their affiliated organizations, or those of the publisher, the editors and the reviewers. Any product that may be evaluated in this article, or claim that may be made by its manufacturer, is not guaranteed or endorsed by the publisher.
