# Supplementary figures and images for: Case Report: TNF-Alpha Inhibitors to Rescue Pregnancy in Women With Potential Pregnancy Loss: A Report of Ten Cases
Source: Front Immunol. 2022 May 25;13:900537. doi: 10.3389/fimmu.2022.900537 (PMC9174430; doi:10.3389/fimmu.2022.900537)

## Slide 1
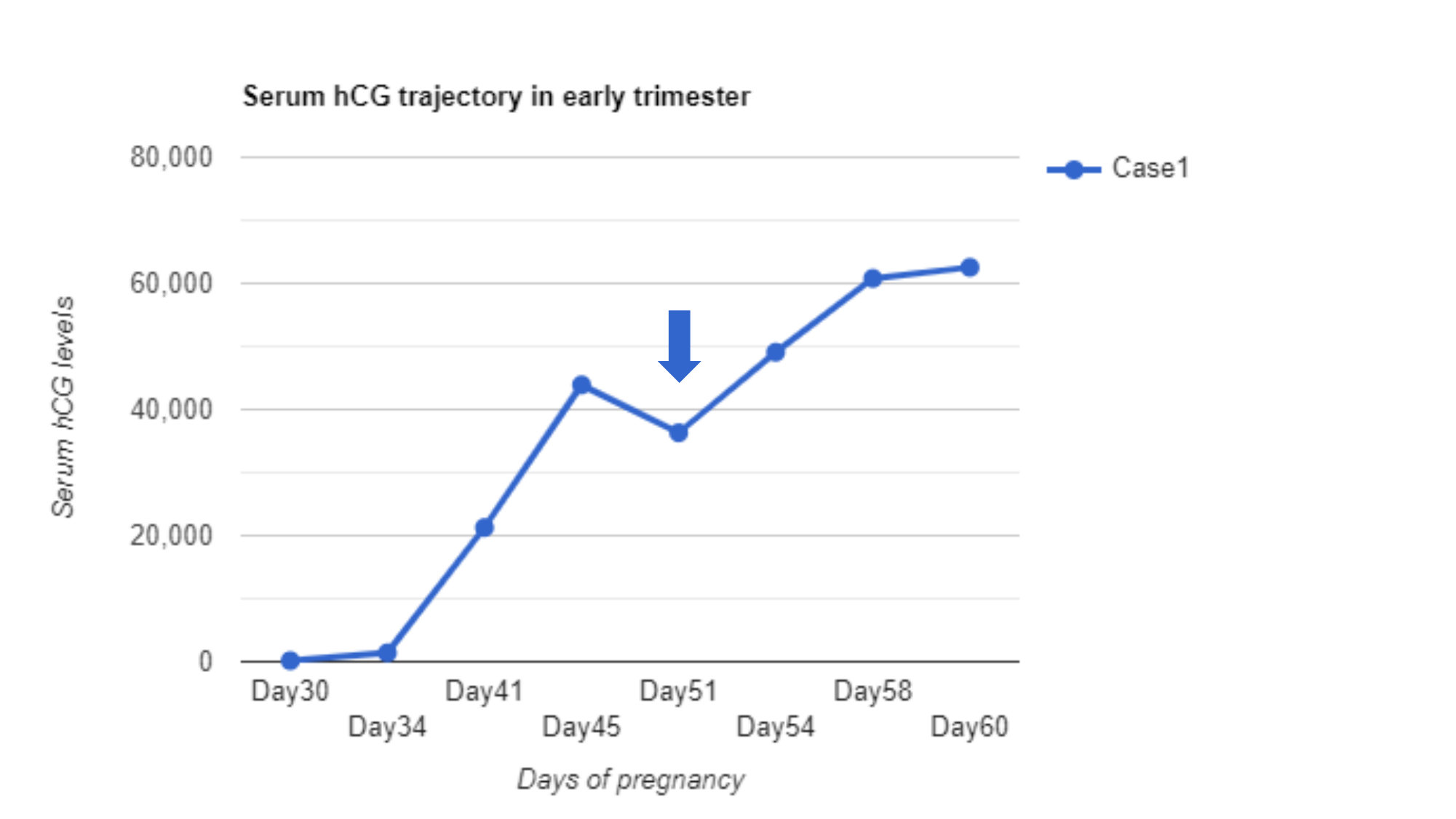

## Slide 2
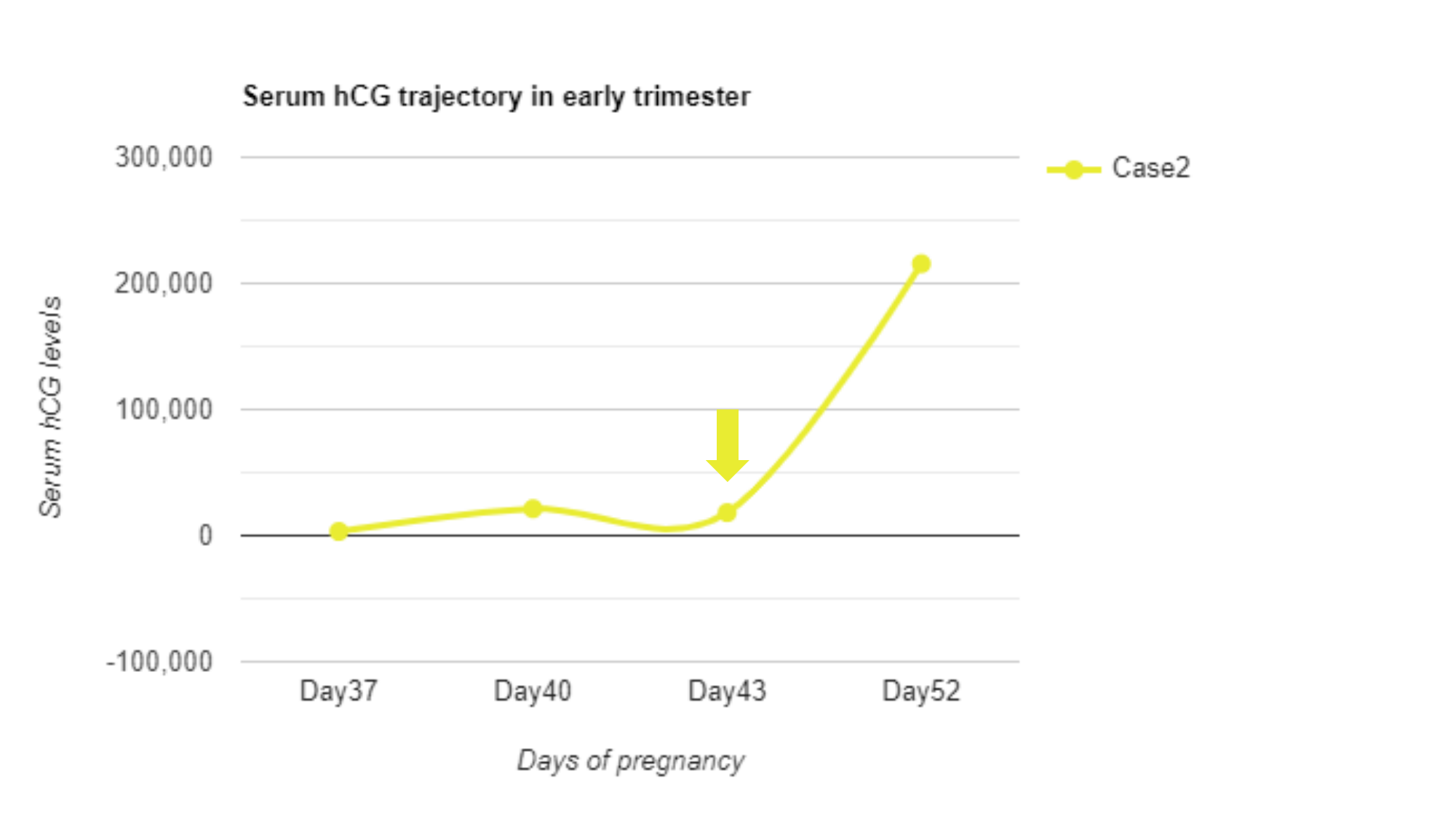

## Slide 3
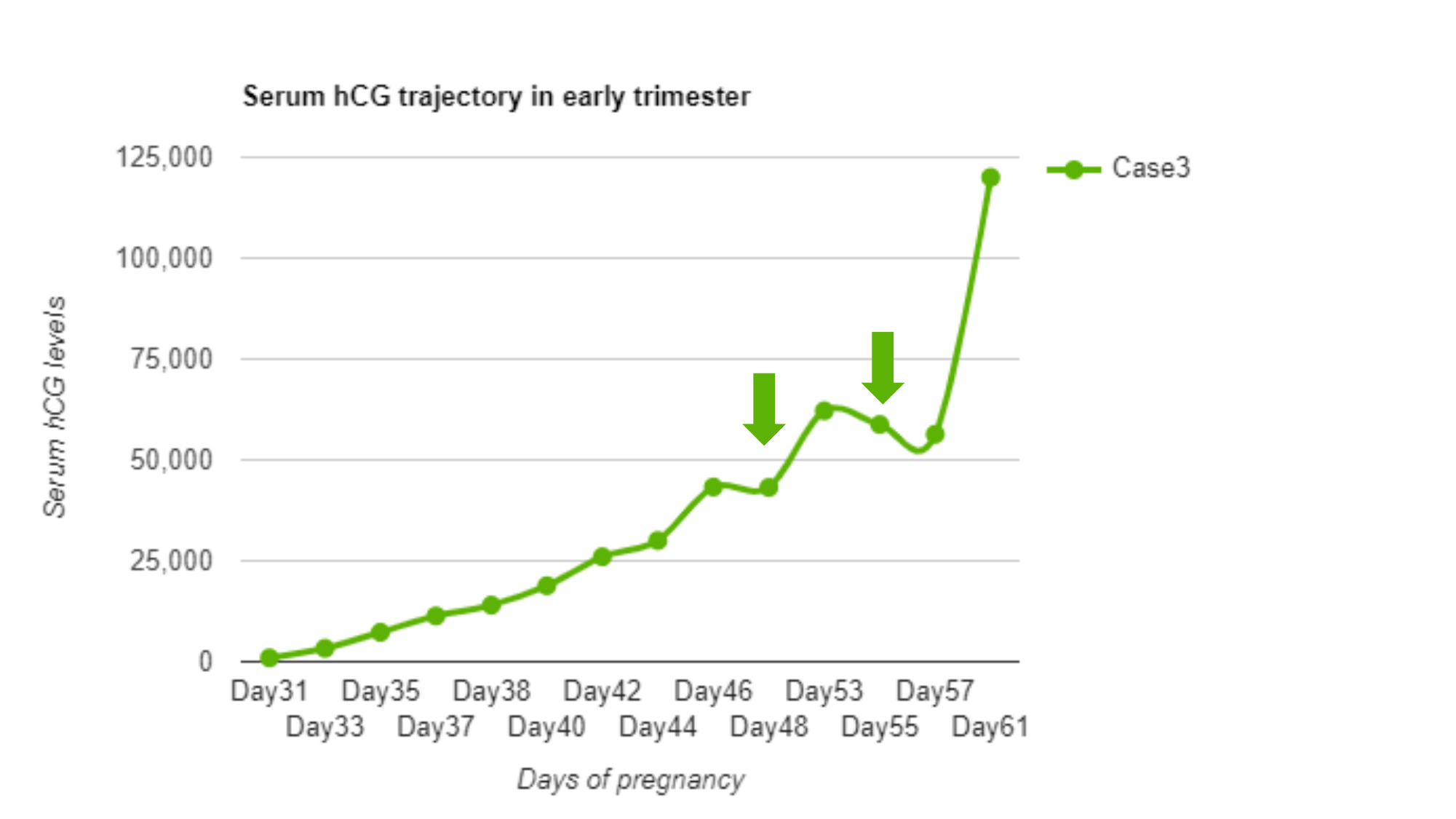

## Slide 4
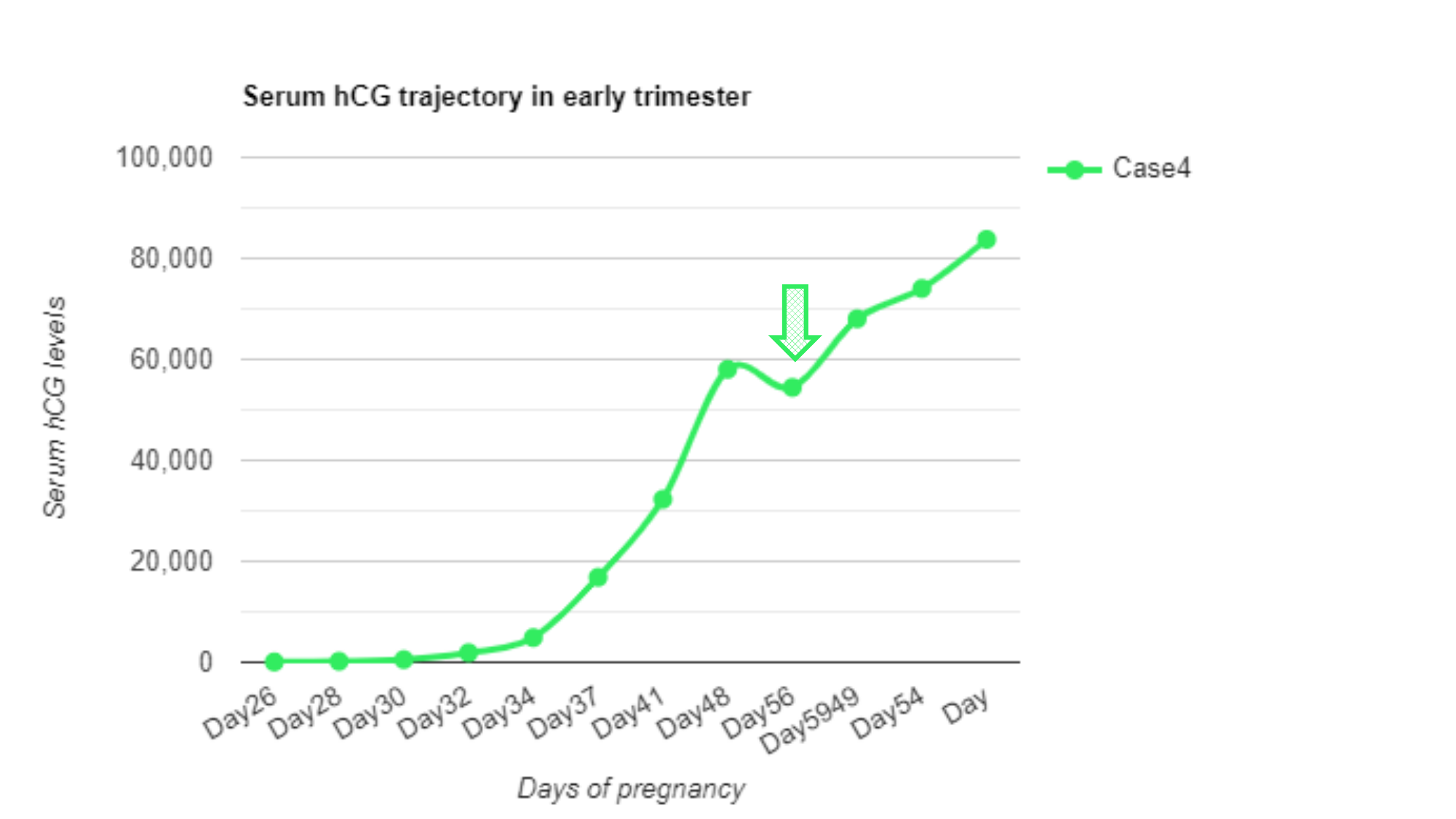

## Slide 5
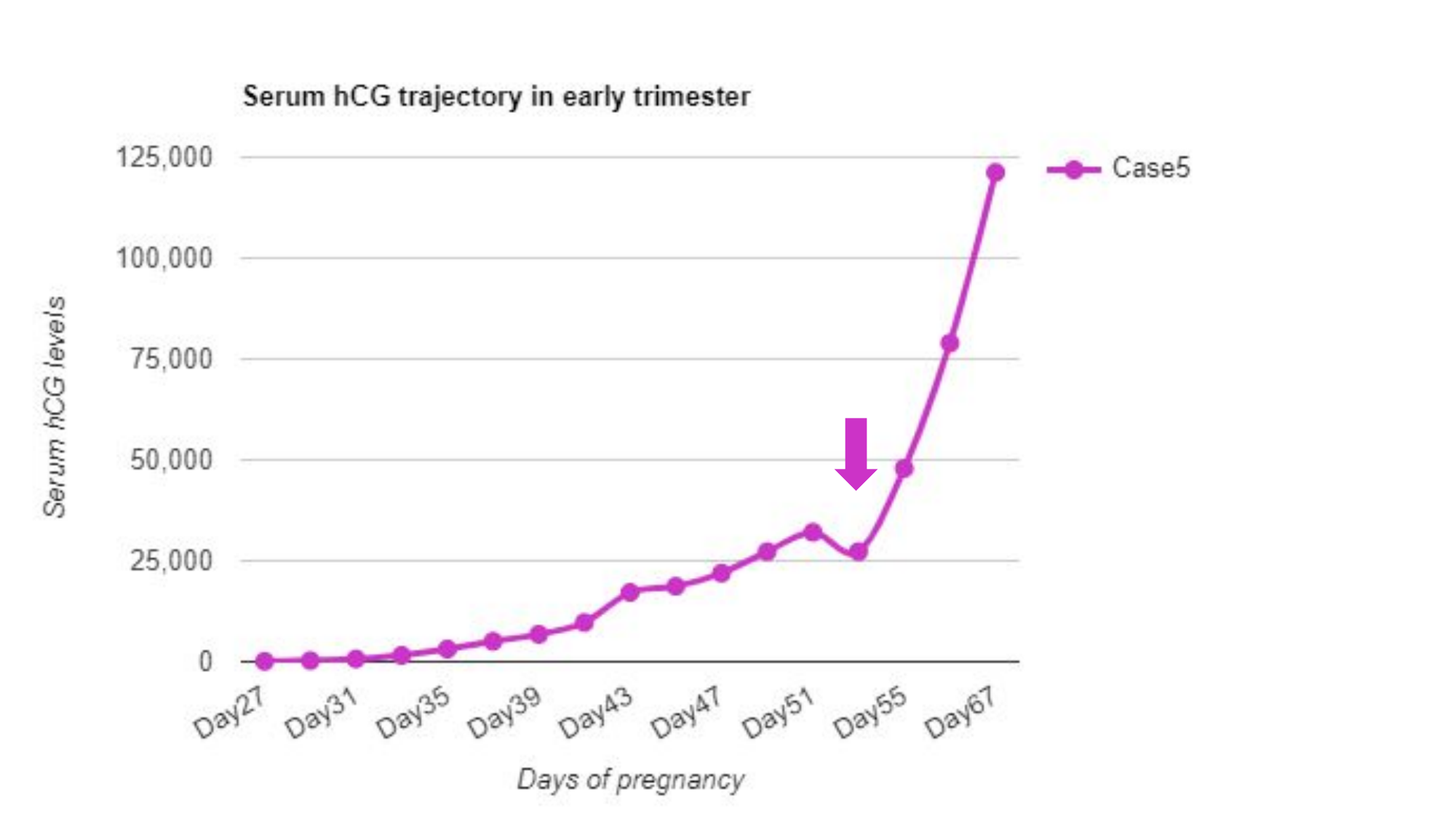

## Slide 6
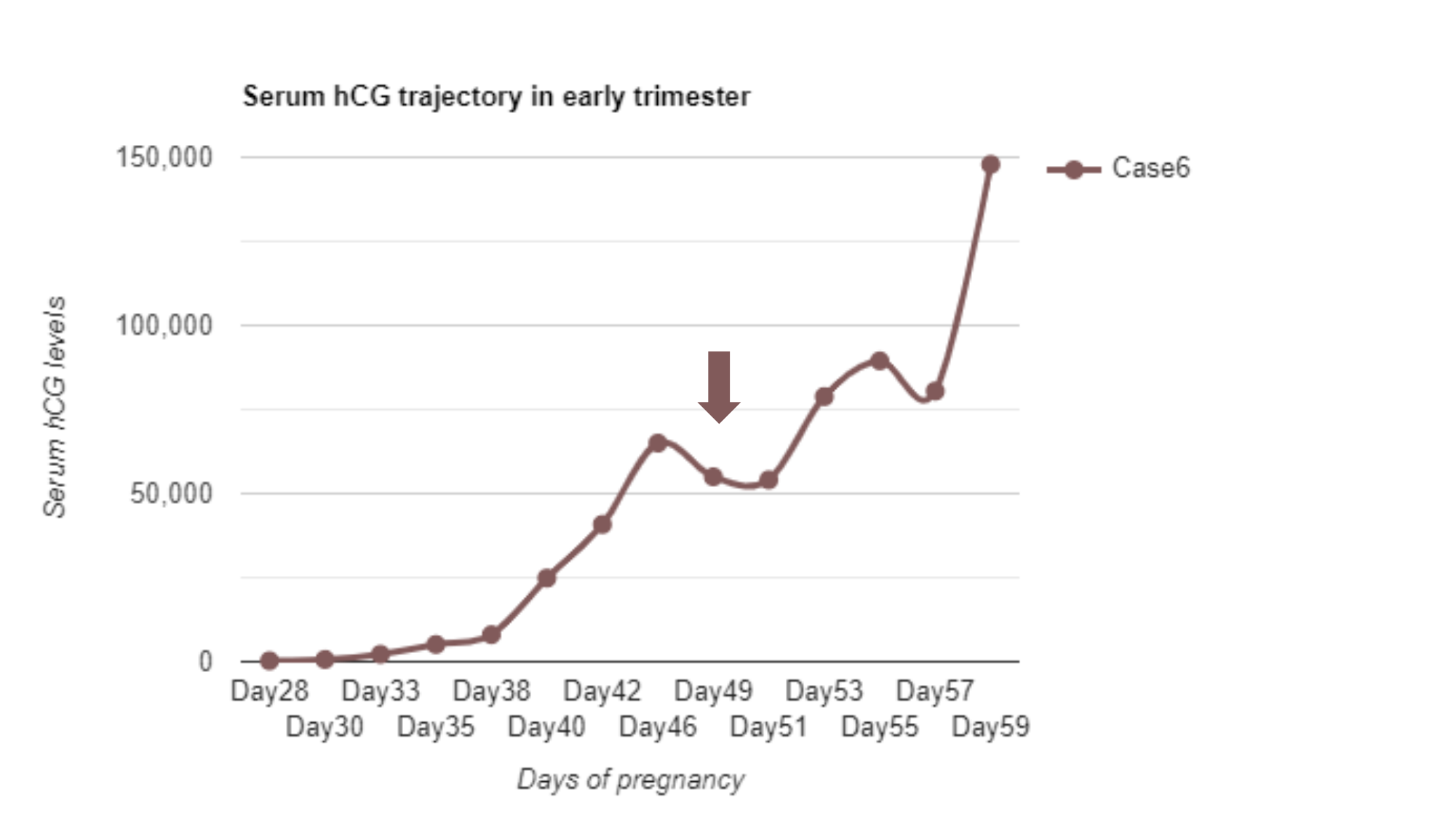

## Slide 7
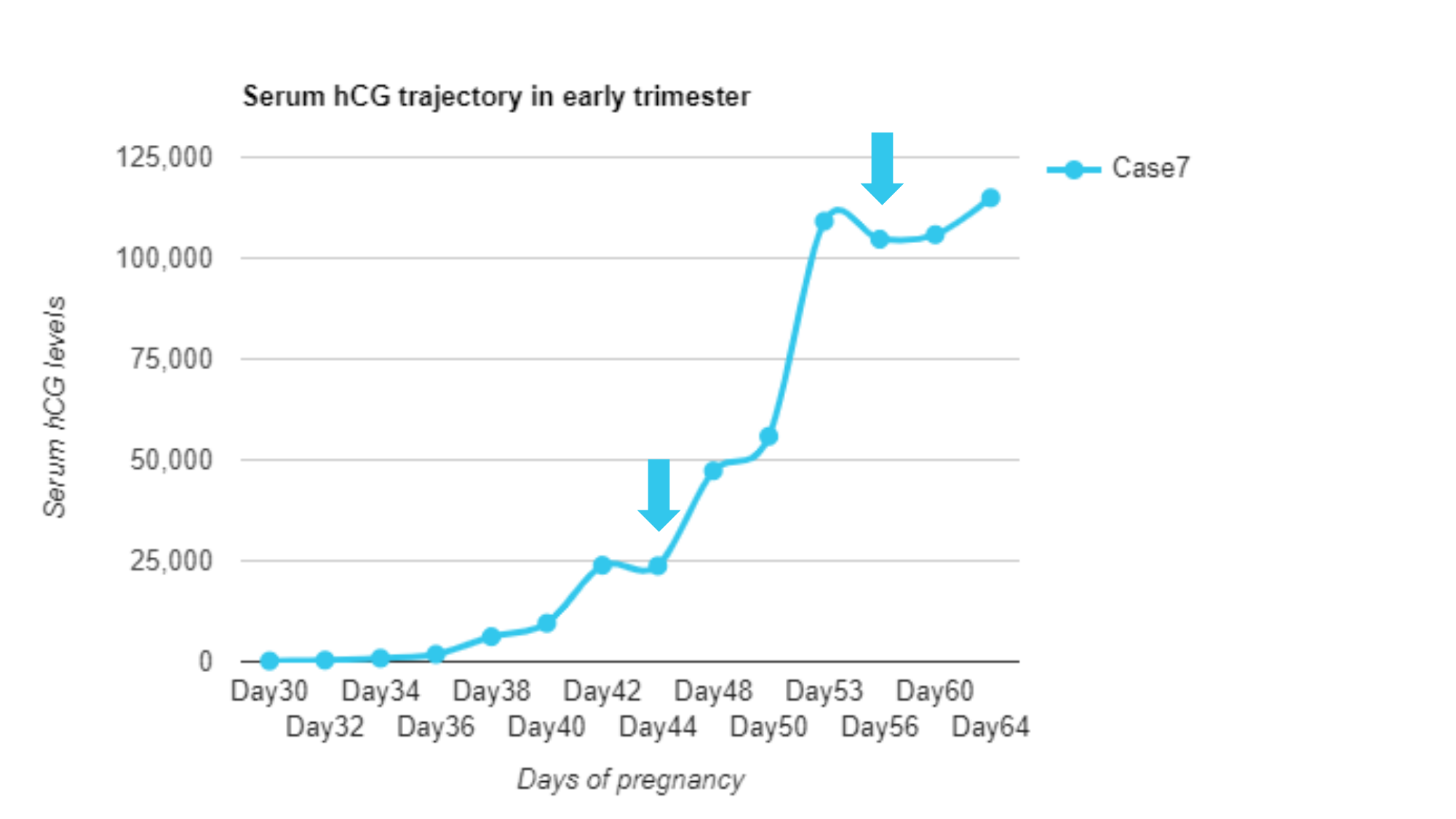

## Slide 8
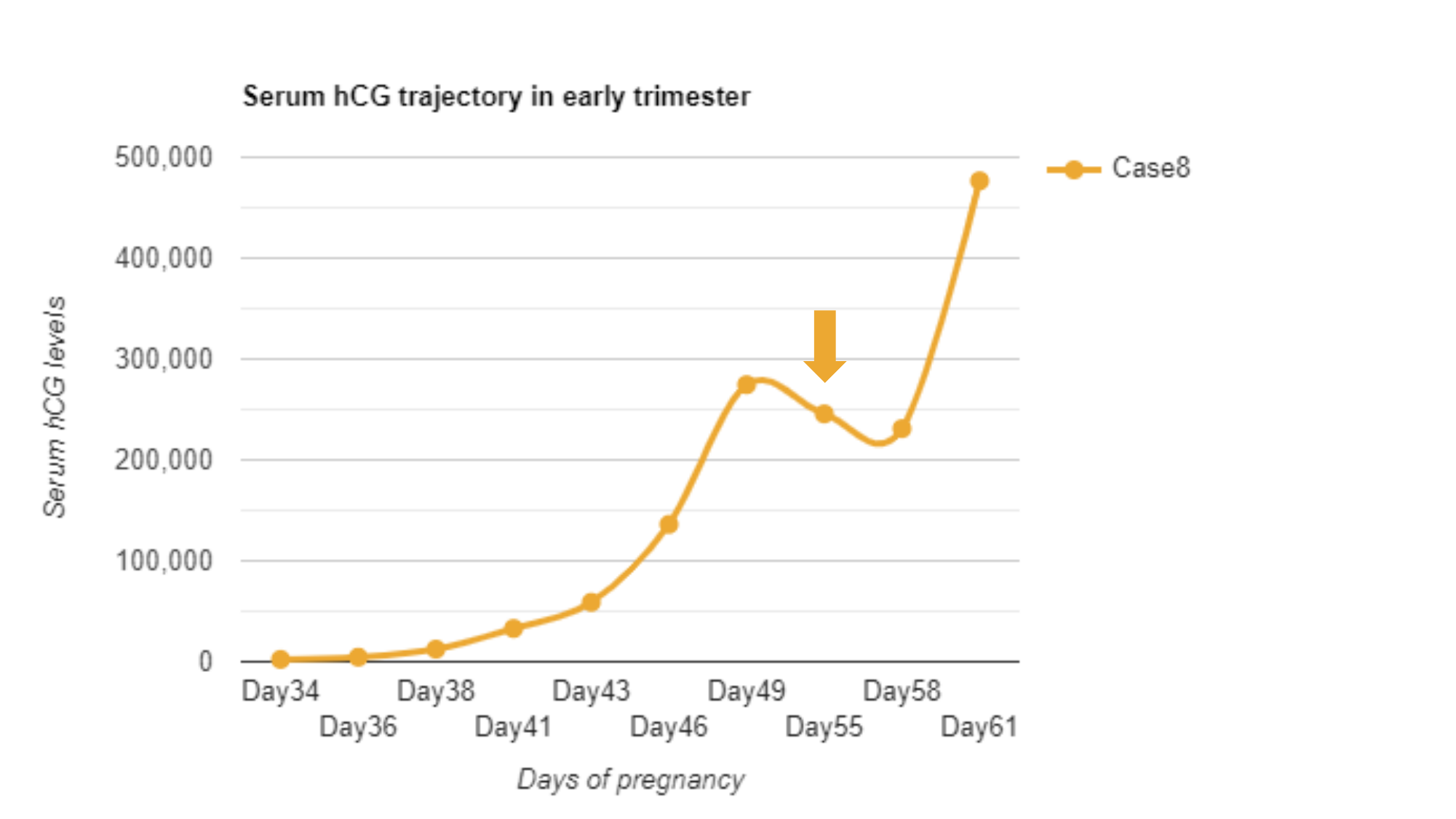

## Slide 9
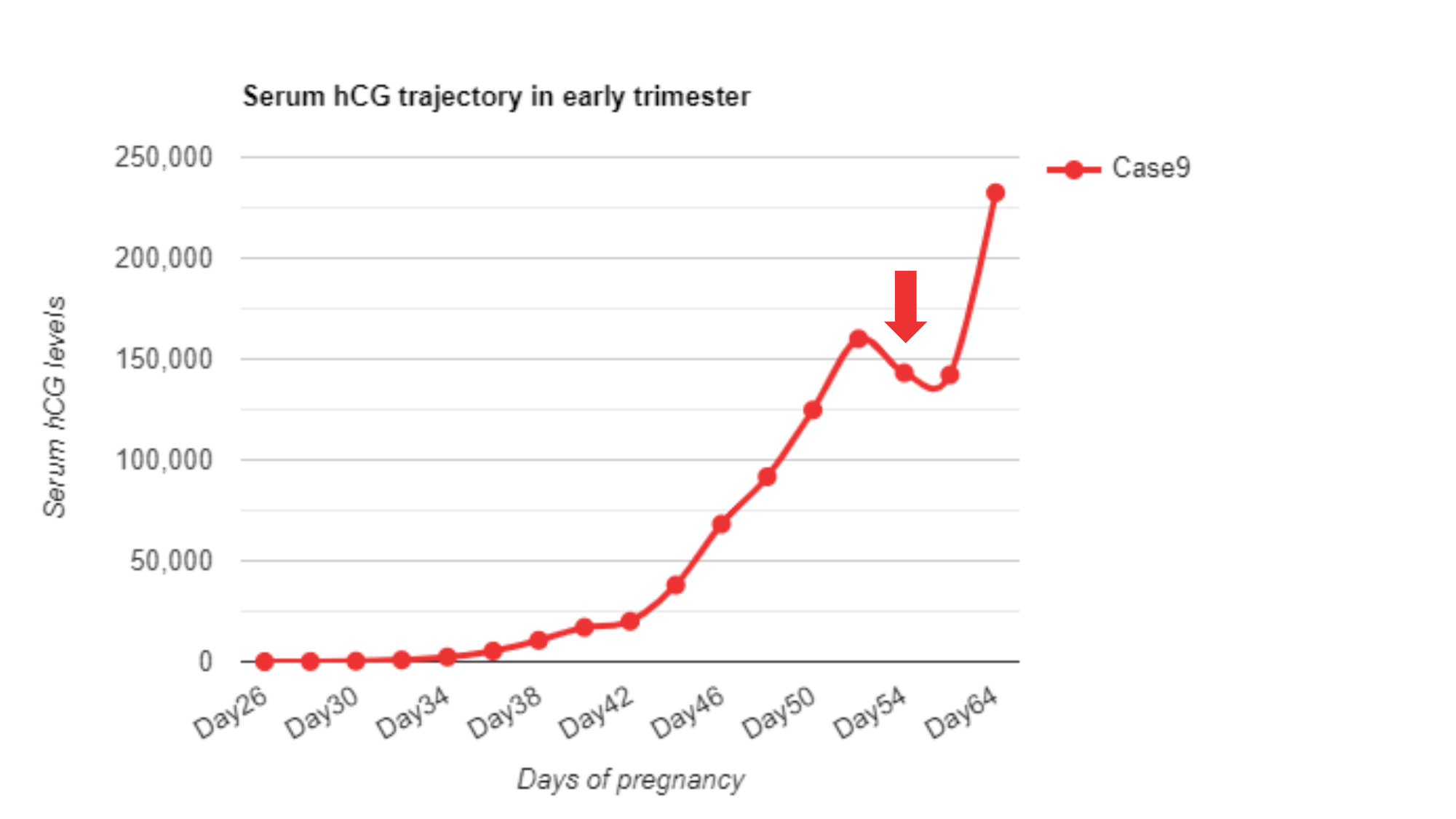

## Slide 10
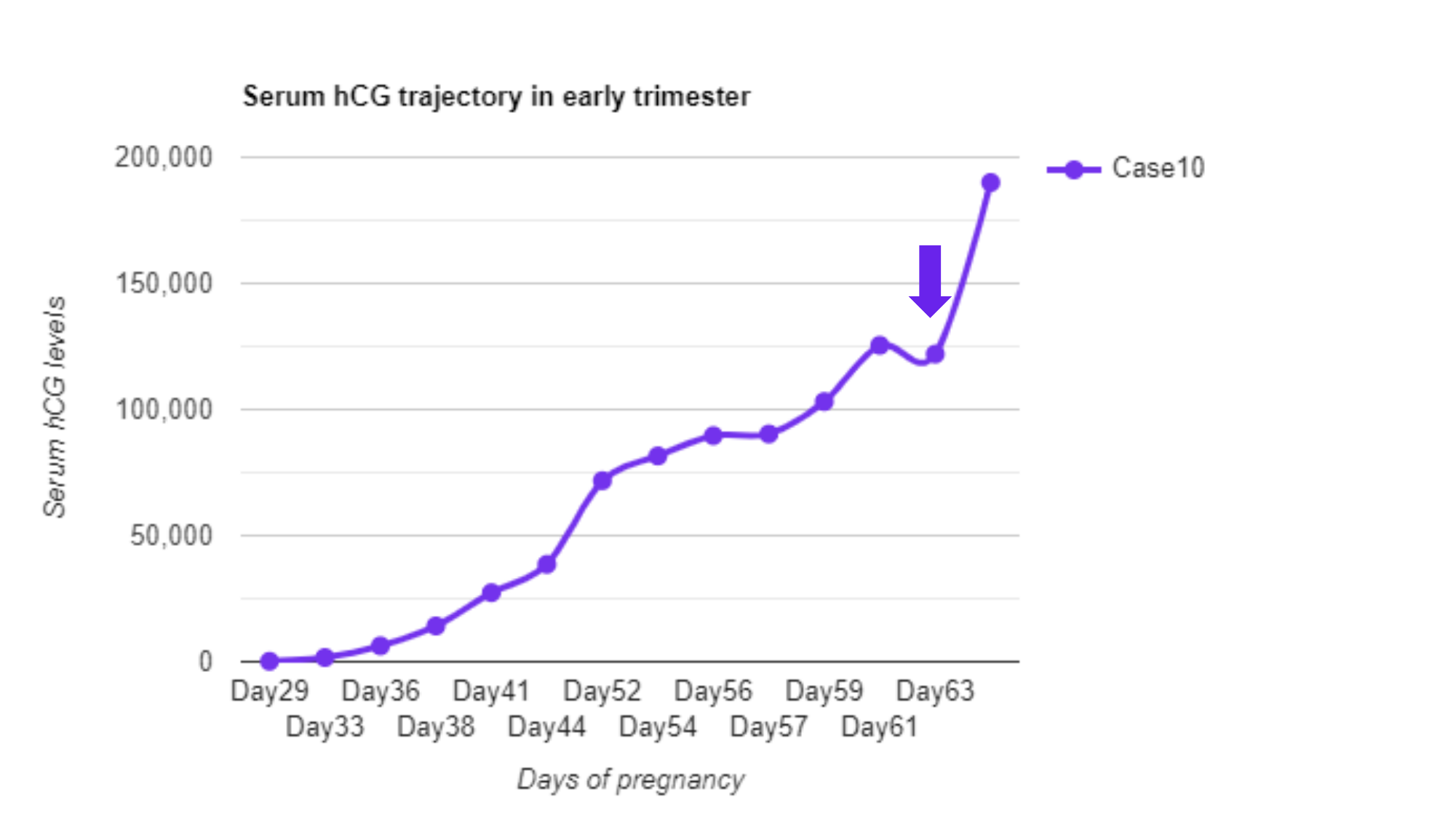

Supplement: Supplementary file 1 [file Presentation_1.pptx]
